# Interfaces, Circuits and Humidifiers

**DOI:** 10.3389/fped.2020.557301

**Published:** 2020-12-07

**Authors:** Rosario Ferreira

**Affiliations:** Pediatric Pulmonology Unit, Department of Pediatrics, Santa Maria Hospital, Academic Medical Centre of Lisbon, Lisbon, Portugal

**Keywords:** non-invasive ventilation, pediatrics, interfaces, circuits, humidification, adverse events

## Abstract

Long-term non-invasive ventilation (LTNIV) has been increasingly used in children to manage chronic respiratory failure and airway obstruction. Interfaces are of paramount importance for non-invasive ventilation (NIV) effectiveness and patient compliance. However, historically, the choice of pediatric mask has been limited by the scarce availability of commercial interfaces. In recent years, an increasing number of different masks have been commercialized for children, allowing to increase the number of patients who could benefit from LTNIV. Factors such as the age of the child, disease, craniofacial conformation, type of ventilator and mode of ventilation, and children's and family's preferences should be taken into account when selecting the appropriate mask. Adverse events such as skin lesions, facial growth impairment, and leaks must be prevented and promptly corrected. Humidification is a controversial issue on NIV, but it may be useful in certain circumstances. Regular cleaning and disinfection of interfaces and equipment must be addressed. During follow-up, educational programs, close supervision, and continuous support to children and families are crucial to the success of LTNIV therapy.

## Introduction

Long-term non-invasive ventilation (LTNIV) has been increasingly prescribed for the management of respiratory complications in children with a wide range of clinical conditions like obstructive, chest wall, neuromuscular, central nervous system, and chronic lung disorders (1–3).

Opposite to invasive ventilation, non-invasive ventilation (NIV) provides respiratory support through the use of a mask, thus avoiding tracheal intubation or tracheostomy. Interface choice is of paramount importance when initiating LTNIV, since the presence of leaks or discomfort may affect the efficacy of LTNIV and patients' adherence to treatment. Over the last years, an increasing number of industrial interfaces have become available for infants and children, with adapted mask and headgear design and ergonomics, and the use of more appropriate materials (4, 5).

When starting any children on LTNIV, different types and sizes of masks should be available to allow the selection of the most appropriate interface. Studies comparing interfaces' performance and comfort are scarce in adults and even more in children, with most of the published studies reporting bench data. Nevertheless, the choice of the interface is also associated with local commercial availability, respiratory care center's experience, and child and family preferences.

Finally, few reviews addressed circuits' and humidifiers' characteristics and indications. The objective of this review is to be a practical and comprehensive reference to the available masks, circuits, and humidification options in LTNIV.

## Interfaces

Interfaces influence compliance, comfort, synchrony, and ventilators' performance (6, 7). An ideal interface is lightweight, is stable, is non-traumatic, has minimal dead space, is durable, has low resistance to airflow, is available in several sizes, is easy to clean and disinfect, is connectable with any ventilator, is easy to take off in order to avoid aspiration if the ventilator crashes or the patient vomits, and is affordable (8, 9).

Interfaces may be vented, if they have holes to guarantee intentional leaks when a single-limb circuit is used. In this case, expiration occurs through the mask, and a minimal expiratory pressure is required to ensure CO_2_ removal from the circuit. Non-vented interfaces, without intentional leak, may be used with double-limb circuits or with a single-limb circuit with an exhalation valve (4). In this case, expiration occurs through the expiratory limb of the circuit or through the exhalation valve, and no minimal expiratory pressure is required.

Interfaces can cover the nose (nasal mask), the nose and the mouth (oronasal mask), the face (total face mask), and in certain particular situations, the mouth only (mouthpiece). Total facial masks, which covers the nose, mouth, and eyes, may be used in particular conditions such as complex craniofacial malformations but are rarely prescribed to home ventilation because of the increased risk of aspiration with emesis, in particular in younger children (10). Nasal pillows (or prongs or cannulas) are minimal contact interfaces, available for children older than 5–7 years. Table 1 summarizes the characteristics, advantages, and disadvantages of different masks.

**Table 1:** Advantages and disadvantages of interfaces.

**Interface**	**Characteristics**	**Advantages**	**Disadvantages**
Nasal mask	Flow through the nose	Allows eating, speaking, secretion management, and using pacifier Several pediatric and infant models and sizes available No risk of aspiration	Risk of: • Nasal dryness and irritation • Mouth leaks • Skin lesion over the nasal bridge and face • Midfacial hypoplasia • Eye irritation in case of leaks
Oronasal mask	Flow through the mouth and nose	No mouth leaks Less risk of midfacial hypoplasia	Limit eating, speaking, secretion management No use of pacifier Risk of: • Aspiration • Suffocation • Skin lesion over the nasal bridge and face • Eye irritation in case of leaks Claustrophobia Few pediatric sizes available
Full facial mask	Flow through the mouth and nose	No mouth leak Less risk of: Facial growth impairmentSkin lesion	Limit eating, speaking, secretion management Risk of: • Aspiration • Suffocation • Claustrophobia Few pediatric sizes available Increased dead space Limited domiciliary indications
Nasal pillow	Flow through the nose	Minimal facial contact Allows eating, speaking, and secretion management Less risk of skin lesionNo risk of aspiration	Risk of: • Nasal symptoms • Nostrils pain and lesions • Mouth leak No pediatric sizes available
Mouthpiece	Flow through the mouth	Allows eating, speaking, and secretion management Allows air stacking No need of headgear No risk of: • Skin lesion • Aspiration • Nasal symptoms • Claustrophobia Better appearance	Needs a collaborative patient Needs capacity of lips seal Risk of: • Mouth and nose leak • Orthodontic problems • Gastric dilatation • Increased salivation • Vomiting

Nasal masks are the most used interfaces in both pediatric and adult patients (3, 5, 11). The risk of aspiration is lower, because the mouth is not covered by the mask. Mouth leaks may be avoided by using a pacifier in infants or chin straps in older children (12). Speaking is possible, while eating and drinking should be carefully balanced against the risk of aspiration, especially with bilevel ventilation.

Oronasal masks may be indicated in children who do not tolerate nasal masks or who have important mouth leaks, but limited availability of commercial masks restricts their use to older children (5). The use of oronasal masks limits speech and oral feeding capacity, and there is an increased risk of aspiration if children vomit or in case of ventilator failure. Moreover, in adults with obstructive sleep apnea, the use of oronasal interfaces may worsen upper airway obstruction, thus leading to the need of higher pressures (13).

Nasal pillows or prongs are inserted into the nostrils, and the pressure generated by the continuous positive airway pressure (CPAP) device helps to seal the soft material against the nose inner walls (14). Like nasal masks, they allow the child to eat and speak and have reduced risk of suffocation by vomiting or ventilator failure. Pediatric sizes are not commercially available, so only older children and adolescents are candidates for this type of interface (15). In older children, a trend for better treatment adherence was found for this kind of interface, when compared with nasal mask (16).

Mouthpiece is indicated in patients who need daytime ventilation, mostly neuromuscular patients (17). The patient receives respiratory support through a mouthpiece supported by a flexible arm kept near his/her lips. The patients must be cooperative and have sufficient muscular strength to seal their lips around the mouthpiece (18). Respiratory support is triggered when the patients places their lips in the mouthpiece, creating a small inspiratory pressure or a sip. Some ventilators have a mouthpiece dedicated mode and a very sensitive trigger, which allows the patient to demand respiratory support with minimal effort (19). Angled mouthpiece, the most frequently of 15 or 22 mm, is most commonly used, as it is easier for the patient to grasp (20).

Mouthpiece interface allows the patient to eat and speak and has no risk of skin breakdown (19). Patients on this mode of ventilation can independently perform an air stacking maneuver, which increases patient's peak cough flow and improves airway secretions drainage (20). Mouthpiece ventilation is used during wakefulness, but there are few reports describing the use during sleep with a lip-seal apparatus that keeps the interface secure (21). Adverse events of mouthpiece have been described, such as increased salivation, orthodontic problems, gastric distention caused by swallowed air, and nose leaks (19, 20). In case of nose leaks, nasal clips may be used. Mouthpiece ventilation is not possible in case of poor cooperation or in case of severe bulbar dysfunction (19).

Customized masks, available in some specialized centers with the appropriate expertise, may be useful, especially for children under 2 years, and with craniofacial malformations. These interfaces are described as more comfortable, leading to less skin lesions and having the potential to increase ventilation efficacy (22–24). The growing availability of 3D medical printing leads to the development of 3D-printed custom masks for adults and children (25, 26).

Some authors report the use of modified nasal cannula for non-compliant children using CPAP (27).

Finally, headgear should be considered as important as the mask. A well-fitted headgear maintains the mask in place with restraining straps made of soft material that allows sweating (10), and it is usually attached by adhesive strips, hooks, or magnetic pieces, which are easy to manipulate. Straps must be sufficiently tightened to prevent air leaks but with caution, in order to avoid pressure-related skin lesions and disturbed facial growth (23).

### Adverse Events

Adverse events related to interfaces are common and may have serious consequences and compromise adherence and ventilation efficacy (5, 23). Changing the interface should be considered if there is any evidence of discomfort, skin lesions, high unintentional leaks, or disturbed facial growth (28). The most frequent adverse events and suggested corrective measures are summarized in Table 2.

**Table 2:** Interface-related adverse events and suggested correction measures.

**Adverse events**	**Suggested correction measures**
Nasal, eye, and mouth symptoms	Check mask fit Consider topical nasal treatment (nasal rinse, corticosteroids, and decongestants) Use a humidifier Avoid mouth leak: • Change to an oronasal mask (in patients who are able to take the mask off) • Use chin straps • Use a pacifier, in infants
Air leaks	Check mask size, fit, and integrity Check headgear fit and integrity (customize it, if necessary) If mouth leak: • Change to an oronasal mask (in patients who are able to take the mask off) • Use chin straps • Use a pacifier in infants Mouthpiece ventilation: • Use a nasal clip • Use a lip seal
Skin breakdown	Check mask position, size, fit, and integrity Alleviate mask pressure Hold the tube to diminish movement Re-assess the need of humidification or reduce it Change interface Rotate interfaces Apply hydrocolloid wound care dresses
Midfacial hypoplasia	Alleviate mask pressure Change interface Reduce ventilation hours

#### Nasal, Eye, and Oral Symptoms

Eye symptoms such as discomfort or redness may occur with mask leaks. Mask fitness should be checked and the interface repositioned (5).

Dryness of nose and mouth is a frequent complain during NIV (29), and it is often associated with mouth leaks (30). When mouth leaks occur, nasal function of heating and humidifying the airflow is overcome by the unidirectional flow, resulting in nasal tissue inflammation and increased nasal resistance, leading to patient discomfort (31). Decreasing mouth leaks will alleviate the symptoms. The use of a humidifier reduces nasal discomfort and promotes nasal respiration (32).

Nasal topical treatments such as nasal rinses, topical corticosteroids, or decongestants have been described as being effective in controlling symptoms (5), but there is no clear evidence to support it.

#### Air Leak

Unintentional leaks, occurring between the face and the mask or through the mouth, may have a deleterious effect on NIV and CPAP treatment (33). They may contribute to patient's ventilator asynchrony, autocycling, and inefficient pressurization and volume delivery, all leading to ventilation and sleep disturbance (34, 35) Domiciliary ventilators are designed to compensate for leaks, but their performance depends on the ventilatory modes and the volume of leaks. Different ventilators have shown considerable variation in their ability to compensate leaks, particularly in pediatric patients (36). Bench studies have also shown that dynamic leaks interfere with tidal volume and leak estimation of the ventilators' monitoring software (37).

Mouth leaks lead to mucosal dryness and increased nasal resistance due to reduced humidification, disturbed ciliary activity, and increased mucus secretion (30). These in turn generate a vicious cycle and further sustain mouth leaks. As a consequence, ventilation performance is compromised, treatment fails to provide relief, and treatment adherence is reduced (30).

A first step in controlling leaks is to assess the mask fitting, looking for mal-positioning or deterioration. It may be useful to ask the patient or the caregiver to put the mask in the presence of one member of the health-care team in order to check the correct fitting. The headgear should also be inspected for integrity or strap laxity and be substituted if necessary. In case of mouth leaks, using a pacifier in infants and a chin strap in older children or switching to an oronasal mask may resolve the problem (38). The inclusion of a humidifier in the circuit may help in decreasing nasal resistance and mouth breathing, thus attaining more comfort (39).

#### Skin Breakdown

Skin lesions are frequent adverse events associated with NIV masks (9). Since impermeable silicone polymers are the most frequently used contact materials, increased humidity can provoke skin maceration and, consequently, increased risk of cutaneous lesions (8).

A pressure ulcer (PU) is defined as a local of skin lesion due to pressure, shear, or friction (40). Medical device- related PU results from the use of devices designed and applied for diagnostic or therapeutic purposes (41). PU is more frequent in locations with less subcutaneous tissue like the cheeks, chin, forehead, and nasal bridge (42).

Excessive pressure exerted by the mask, causing tissue ischemia, is the most important risk factor for skin and facial side effects (43). Besides the mechanical effect of pressure associated with straps tension, an increase in inflammatory cytokines in affected skin has been shown (43). An increase in temperature and humidity detected below the mask eases skin maceration and potentiates mechanical effect of friction, contributing to the development of PUs (43, 44). Skin lesions may be further aggravated by poor nutrition and the loss of muscular mass, making neuromuscular patients more susceptible to this kind of complications (34). The humidification of NIV circuits may also contribute to skin lesions by increasing skin moisture (9).

In pediatric patients, the risk factors for PUs are skin immaturity, decreased/reduced mobility, altered neurological status, and the use of masks that are too small or do not fit to the facial anatomy of the child, as what happens in children with craniofacial anomalies (44, 45).

Use of protective dressings between the skin and the mask is common but controversial in literature. In adult patients, it was shown that hydrocolloid materials, which adhere to the skin, increase mask adherence and reduce friction, have a protective effect on mask skin lesions, and contribute to improvements in temperature control and skin barrier function (40). A meta-analysis of 22 studies, three of them included pediatric patients, has shown that hydrocolloid dresses were significantly more effective in preventing PU than gauze or standard skin care (46). The use of these materials as soon as skin redness appears is advised (9). However, not all types of protective patches were shown to prevent skin injury. Therefore, selection of the most suitable mask and headgear, fixation with an optimal tension, and frequent inspection for skin alterations represent key elements for effective PU prevention (45).

The risk of PU may be reduced by appropriate mask type and size selection and by changing or alternating interfaces with different pressure points. Adequate tightness of headgear strains may be checked by passing one finger between the face and the headgear straps in each side of the face (34).

LTNIV health-care team should educate patients and caregivers to pay careful attention to skin integrity in order for early identification of any skin breakdown and take immediate actions to prevent severe lesions.

#### Midface Hypoplasia

Midface hypoplasia is a motive of concern in pediatric LTNIV because continuous pressure over the face in a growing child leads to the molding of the underlying structures and alterations in face development, which may cause airway narrowing and thus aggravate obstructive sleep apnea (23, 47).

In a series of 40 children, facial flattening was identified in 68% patients (23). No correlation was found between facial flattening and age, type of mask, or daily ventilation, but it was more frequent in obstructive sleep apnea and neuromuscular patients who were younger and whose daily ventilator support requirements were longer. A retrospective study in children with craniofacial malformations requiring LTNIV for obstructive sleep apnea, comparing characteristics of compliant and non-compliant patients, found that compliant children showed more facial growth restriction (47).

Changing the interface periodically, alternating pressure points, alleviating the pressure of the headgear (without compromising mask-face seal), and reducing the number of ventilation hours may help to reduce facial growth restriction (23, 38).

### Select an Interface

Interface choice depends on several factors, including age and neurocognitive development, craniofacial anatomy, specific disease's characteristics, severity of respiratory disturbance, selected ventilator, health team experience, and child's and caregiver's preference (5, 11). When choosing and fitting an interface, ample care should be taken to ensure patient's familiarity with the equipment and comfort, since prompt symptom relief and comfort increase adherence to treatment (48).

#### What are the steps to select a mask?

- Take some time with the child and caregivers to explain the ventilation therapy, objectives, indications, and contraindications.
- Consider the age, disease, type of ventilation, ventilator, and circuit to select ventilated or non-ventilated masks. Nasal masks are the most used interface in pediatric patients. Infants and younger children are essentially nasal breathers, and most of commercially available pediatric masks are actually nasal interfaces (49). The use of oronasal masks and nasal pillows is limited by the limited availability of pediatric sizes. Mouthpiece ventilation may be prescribed to older and cooperative children who need daytime ventilation.
- Have several masks available to test the most adequate one. Consider the child's and caregivers' preferences in the decision process.
- Evaluate the headgear: in small children or children with craniofacial anomalies, some adjustments may be necessary.
- Initiate ventilation to evaluate leaks and mask fitness. Identify pressure points and anticipate measures in order to prevent skin lesions.
- In anxious or young children, as well as in those with neurocognitive disability, it may be important to let the child take the mask home some days before initiating ventilation, for familiarization with the equipment.
- Be prepared to change the interface if needed, especially in the first days of treatment.

Behavioral therapy has been shown to be effective in increasing interface tolerance in children not compliant (50), especially in younger and neurocognitive-compromised patients (51). Some anecdotal reports describe the use of medical hypnosis as a valuable tool to reduce anxiety in children and parents (52).

## Circuits

Circuits are tubing systems that connect the ventilator to the interface and have the role to deliver positive air pressure to the patient's airways, allowing clearance of exhaled air. There are three types of circuits. The double-limb circuit consists of an inspiratory tube and an expiratory tube with inspiratory and expiratory valves at the proximal end and a Y-shaped ending at the distal end just proximal to the interface. The single-limb circuit consists of a single tube. Since such an assembly could cause mixing of the inspiratory and expiratory air and consequently leading to CO_2_ rebreathing, an expiratory valve or an intentional leakage system is incorporated into the circuit. The former is termed “non-vented” and the latter “vented” circuit. In the first case, the expired air leaves the tube through an expiratory valve positioned at the circuit's distal end. In the second assembly, an intentional leak at the distal end of the tube or in the interface itself allows for adequate exhaled air washout (11, 53).

A double-limb circuit, a non-vented single-limb circuit with a distal expiratory valve, or a vented single-limb circuit can be used in combination with non-vented masks. Currently, the large majority of masks and prongs are of the vented type. Holes in masks are more effective than exhalation valves to prevent CO_2_ rebreathing (54). If zero positive end-expiratory pressure (PEEP) condition is required, only a double-limb circuit or a single-limb circuit with an actively driven exhalation valve can be used.

## Humidifiers

NIV provides unidirectional airflow, often at high flow rates. Even though nasal interfaces are predominantly used and consequently the air tempering actions of the nasal airway is preserved, such artificial conditions often cause nasal irritation, increase nasal resistance, compromise mucociliary clearance, reduce tidal volume, and finally, compromise NIV compliance (55, 56).

Heated humidification decreases nasal resistance and its deleterious effect on tidal volume, it increases patient comfort and adhesion to NIV (56), and it has been advised for improvement in compliance to CPAP in adult patients (57).

Humidifiers may be interposed in the ventilatory circuit, when indicated. Mechanical ventilation utilizes two types of humidification. Active humidification consists of a heated humidifier, which requires an external source of heat and water. Alternatively, passive humidification is attained by a heat and moisture exchanger (HME), interpositioned into the respiratory circuit at the location where heat and humidity of the air exhaled can be caught and subsequently passed to the next inhalation (32). The first heating modality is often preferred in LTNIV, as it can provide smaller dead space, reduce the work of breathing, and also be effective in air conditioning (55). Heated humidifiers may be internal (built into the ventilator) or external (connected to the ventilator by tubing) (58). Internal humidifiers have less impact on ventilator performance (58).

Inhaled air temperature and humidity should be set to the level most comfortable to the patient (39). Since ambient conditions vary, modifications of the humidification settings need to be foreseen. In a cold environment, relatively overheated air may precipitate vapor condensation in the tubing, thus leading to suboptimal NIV performance. Some ventilators integrate humidifiers combined with heated tubes, which allow automatic control of humidity and air temperature according to ambient variations (59).

Addition of a humidifier may increase NIV comfort and compliance, when patients complain about nose and mouth dryness or irritation. Special care needs to be taken in small children, since additional humidity may increase secretions and thus compromise their airway patency (60).

Water vapor condensation in the tube may be reduced by covering the tubes with a cloth, placing them under the bedding, or using heated circuits with integrated heated wires that have been recently described to contribute to a better sleep quality and adherence to NIV therapy (61).

It needs to be considered that humidifiers may increase the respiratory circuit's resistance and dead space, interfering with ventilator's triggering and pressurization. Therefore, efficacy of provided ventilatory support should be reevaluated in case a humidifying system is installed (56).

## Cleaning and Disinfection

There is a lack of clear guidance on home ventilation equipment cleaning. Dirty circuits and masks are more prone to be contaminated with bacteria, leading to a higher risk of patient's bacterial colonization (62). Nevertheless, a study found that the type of ventilator, total daily time of ventilation, and the presence of humidifier did not seem to be associated with equipment's bacterial contamination (63).

In a pilot study of 52 ventilated patients, which included 12 children, the authors found the cleaning of the masks to be highly variable, reaching an average 2.9 (0–7) times per week, and only rarely to be disinfected. Bacterial contamination was identified in only 16% of participants. The same bacteria were isolated from the mask and sputum in a cystic fibrosis patient (64).

Washing all components twice monthly in the dishwasher has been shown to be effective in cleaning masks and circuits. When not available, hot water and detergent will be sufficient. After washing, every component must be dried in room air (65).

If a patient is particularly susceptible to respiratory infections, disinfection with 0.5% hypochlorite solution may be indicated (65).

## Follow-up

At every follow-up visit, mask fitting, adverse events, and equipment hygiene should be checked and reviewed. Careful attention to the patient and/or the caregiver complaints is crucial in order to increase patient satisfaction and comfort and ultimately the adherence to treatment. The ventilation team should be vigilant and proficient in delivering solutions for inadequate ventilation or patient's discomfort.

## Future Research

In the last few years, research on LTNIV in children has increased, but there are still significant gaps in our knowledge and equipment availability. Although the availability of nasal masks has increased for infants and small children, oronasal masks and nose pillows do not exist for these age groups. Further investigation on mask exerted facial pressure points and newer materials is needed. Advancements in 3D printing of affordable individually customized masks could have an important role in reducing severe adverse events in patients with craniofacial abnormalities. The role of humidification in pediatric LTNIV needs further validation. The impact of different interfaces, circuits, and humidifiers in ventilators' performance in pediatric age also needs to be addressed in future research.

## Conclusion

Correct choice of the interface is essential for ventilatory support efficacy and compliance. Although nasal masks are the most frequently used in pediatric age, there are no guidelines on the choice of an optimal interface. Similarly, the guidance on humidification use in children on LTNIV is scarce. Early and careful attention must be paid to any sign of adverse events, patient's discomfort, or suboptimal ventilation in order to attain the best therapy results.

## Author Contributions

RF has made the literature search and elaborated the manuscript.

## Conflict of Interest

The author declares that the research was conducted in the absence of any commercial or financial relationships that could be construed as a potential conflict of interest.
